# Hepatocellular jaundice with abnormally high CA19-9: A case report

**DOI:** 10.1097/MD.0000000000046994

**Published:** 2026-01-23

**Authors:** Yangqing Cui, Yijing Zhang, Yuxi Huang, Rui Feng, Qunqun Chen

**Affiliations:** a The Third Affiliated Hospital, Guangzhou University of Chinese Medicine, Guangzhou, China.

**Keywords:** CA19-9, hepatocellular jaundice, suspicious cancer

## Abstract

**Rationale:**

Carbohydrate antigen 19-9 (CA19-9) is a mucin-type glycoprotein tumor marker that is present in trace amounts in normal human tissues, including the pancreas, bile ducts, stomach, colon, and salivary gland epithelium. In pancreatic cancer patients, the positive rate of CA19-9 exceeds 80%. Here, we report a case of hepatocellular jaundice accompanied by a marked elevation in CA19-9 levels.

**Patient concerns:**

A 55-year-old female patient was admitted to our hospital with a 6-month history of abdominal distension and dark urine, which had worsened along with scleral jaundice over the past week.

**Diagnoses:**

The initial diagnosis upon admission was “jaundice under investigation.” Laboratory investigations revealed a markedly elevated serum CA19-9 level of 284.8 units per milliliter, raising the possibility of an underlying neoplasm. However, no definite malignant lesions were detected on whole-body positron emission tomography–magnetic resonance imaging. The patient had a long-term history of chronic hepatitis B virus infection with a high viral replication state, suggesting chronic liver injury. Jaundice-related examinations showed elevated levels of total, direct, and indirect bilirubin, markedly abnormal transaminases, and positive urine bilirubin – findings consistent with the biochemical profile of hepatocellular jaundice. Abdominal computed tomography imaging revealed no biliary dilation, ruling out obstructive jaundice. The absence of anemia or abnormal hemolysis-related parameters argued against hemolytic jaundice. Although tumor markers (CA19-9 and alpha-fetoprotein) were transiently elevated, the absence of malignant masses on imaging and the subsequent normalization of CA19-9 levels following treatment led to the exclusion of malignancy-associated jaundice. The final diagnosis was confirmed as hepatocellular jaundice associated with viral hepatitis.

**Interventions:**

The patient received active antiviral therapy, liver-protective treatment, and symptomatic supportive care.

**Outcomes:**

The patient’s jaundice significantly subsided, and liver function parameters gradually improved. The patient was discharged after meeting the relevant criteria. A 6-month follow-up examination showed that CA19-9 levels had essentially returned to the normal range.

**Lessons:**

This case demonstrates a significant and reversible elevation of CA19-9 in benign hepatocellular jaundice, the dynamics of which correlated with disease activity. It serves as a critical reminder that CA19-9 can be a misleading biomarker for malignancy in this clinical scenario.

## 1. Introduction

The paradigm of cancer management has evolved from nonspecific cytotoxic agents toward precision medicine,^[1]^ relying on biomarkers to guide targeted and immunotherapeutic strategies.^[2]^ A pivotal framework categorizes therapies based on their target: cancer cells or the tumor microenvironment. This classification is vital for interpreting biomarker dynamics, as nonmalignant inflammation within the microenvironment can drive significant biomarker fluctuations independently of neoplastic transformation.^[3]^ A prime example is carbohydrate antigen 19-9 (CA19-9), which can be elevated not only due to malignant secretion by pancreaticobiliary tumors but also from inflammatory stimulation^[4,5]^ in benign conditions such as cholangitis^[6]^ and obstructive jaundice.^[7]^ This biological duality creates a diagnostic challenge, necessitating careful differentiation between neoplastic and inflammatory etiologies. The present case of markedly elevated CA19-9 in a setting of hepatitis B virus (HBV)-related hepatocellular jaundice perfectly illustrates this clinical dilemma.

As one of the most clinically valuable tumor markers in the field of digestive system malignancies, CA19-9 has established a well-validated application framework across 3 critical clinical phases, early diagnosis, treatment response monitoring, and prognosis assessment, in cancers such as pancreatic cancer, cholangiocarcinoma, gastric cancer, and colorectal cancer.^[8]^ From the perspective of early diagnosis, early detection remains a central challenge in improving outcomes for patients with digestive system cancers. Serum CA19-9 exhibits a “prewarning” characteristic, as its abnormal elevation often precedes the onset of typical clinical symptoms, such as persistent abdominal pain, unexplained weight loss, and gastrointestinal obstruction, thereby providing quantifiable and actionable biological clues for early screening.^[2]^ CA19-9-based early warning assists in the timely detection of tumor lesions, significantly improves the rate of radical surgical resection, and lays a foundation for enhancing long-term patient prognosis.^[9]^ CA19-9 serves as a dynamic biomarker for monitoring treatment response, reflecting tumor sensitivity to therapeutic interventions. A pronounced decline in serum levels within 3 weeks after surgery, chemotherapy, or radiotherapy typically indicates favorable treatment efficacy. Conversely, persistently elevated or rising CA19-9 levels may indicate drug resistance, disease recurrence, or distant metastasis, such as hepatic metastasis in pancreatic cancer. These dynamics provide an objective basis for clinical decision-making, enabling timely adjustment of treatment strategies, including switching chemotherapeutic agents or incorporating targeted therapies, to avoid ineffective treatment and mitigate disease progression.^[10]^ In prognostic evaluation, CA19-9 serves as an independent predictor of survival in patients with digestive system cancers, with both baseline levels and posttreatment dynamics offering critical prognostic insights. Studies^[11]^ have reported that a baseline CA19-9 level exceeding 370 units per milliliter (U/mL) in pancreatic cancer, or persistent elevation after treatment, is associated with significantly reduced 5-year survival compared with patients with normal or decreasing values. Conversely, a rapid and sustained decline, such as a stepwise reduction over 3 consecutive treatment cycles, has been correlated with improved long-term survival outcomes. Nevertheless, CA19-9 lacks specificity for malignancies of the digestive system, an inherent limitation that complicates its clinical interpretation.

Elevated serum levels of CA19-9 have been documented in various benign conditions, including acute cholecystitis,^[4,5]^ obstructive jaundice,^[7]^ choledocholithiasis, cholangitis, and biliary manipulation.^[6]^ These transient elevations are primarily attributed to biliary obstruction or inflammation-mediated stimulation of the biliary epithelium. Such nonneoplastic elevations of CA19-9 may complicate diagnostic accuracy, increasing the risk of unnecessary additional investigations (e.g., Positron emission tomography–computed tomography scans) or misdiagnosis. To mitigate this limitation, a multidimensional integrated evaluation system should be established in clinical practice. This approach employs a cross-verification strategy combining CA19-9 testing, imaging examinations, assessment of clinical signs and symptoms, and complementary tumor biomarker assays to confirm or rule out malignancy. The value of this model is 2-fold: it spares patients without cancer from unnecessary treatments (such as chemotherapy), thereby preventing associated physical harms (e.g., myelosuppression), psychological distress, and financial burdens; meanwhile, it ensures that cancer patients receive timely and individualized therapy. This aligns closely with the core principle of precision oncology,^[2]^ guiding personalized treatment through multidimensional evidence, and further underscores the clinical relevance and academic validity of the integrated approach. Beyond its role in clinical diagnostics, the dynamic fluctuation patterns of CA19-9, including transient elevations in benign conditions, carry important implications for drug development and clinical trial design. It has been established that CA19-9 serves a dual function in clinical trials: enabling biomarker-driven patient stratification and providing a dynamic indicator of treatment response. By reducing cohort heterogeneity and supporting real-time therapeutic assessment, CA19-9 contributes to accelerating the translation of novel therapies from preclinical research to clinical use. A multidisciplinary approach is critical to mitigating the risk of overinterpreting individual biomarkers. CA19-9 should not be utilized as a standalone indicator but must be evaluated within an integrated framework of disease-specific markers and comprehensive clinical contextualization.^[12]^ For example, infection-induced gastrointestinal inflammation, such as that associated with *Helicobacter pylori* or HBV, has been linked to elevated CA19-9 levels through shared inflammatory pathways, which may confound cancer diagnostic accuracy.^[13]^ This mechanism highlights both the interpretative challenges posed by CA19-9 in complex clinical contexts and reinforces the theoretical imperative for multifactorial and integrated analysis.

Hepatocellular jaundice,^[14]^ a well-recognized benign condition associated with nonneoplastic CA19-9 elevation, underscores the importance of accurate clinical interpretation through its clearly delineated pathophysiology and diagnostic criteria. This form of jaundice arises from hepatocyte injury or dysfunction, induced by factors such as viral hepatitis, alcoholic liver disease, drug-induced liver injury, or autoimmune hepatitis, which impairs bilirubin uptake, conjugation, and excretion. The resultant accumulation of both direct and indirect bilirubin leads to hyperbilirubinemia and clinically evident jaundice. At its core, hepatocellular jaundice represents a disturbance in bilirubin metabolism mediated by hepatic cellular damage. The diagnosis of hepatocellular jaundice necessitates a comprehensive synthesis of clinical, biochemical, imaging, and histopathological evidence. Clinically, patients typically present with jaundice, characterized by a light to golden-yellow discoloration of the skin, mucous membranes, and sclera, accompanied by nonspecific symptoms such as fatigue, anorexia, and right upper quadrant discomfort. An established etiology, such as viral hepatitis, chronic alcohol consumption, or hepatotoxic drug exposure, should be identified. Laboratory studies reveal elevated total bilirubin with proportional rises in both direct and indirect fractions, markedly increased aminotransferase levels (often exceeding 5 times the upper limit of normal), and frequently hypoalbuminemia, prolonged prothrombin time, and elevated bile acids. Imaging modalities, primarily abdominal ultrasound supplemented by CT or magnetic resonance imaging (MRI) when indicated, demonstrate hepatic parenchymal changes in the absence of biliary dilation. In indeterminate cases, percutaneous liver biopsy provides definitive evidence through histopathological findings such as hepatocyte degeneration, necrosis, or inflammatory infiltration. This integrative approach^[15]^ reliably differentiates hepatocellular jaundice from hemolytic or obstructive causes and offers essential insights for distinguishing benign from malignancy-associated CA19-9 elevation.

## 2. Case presentation

All procedures were conducted in line with the ethical standards of the 1964 Helsinki Declaration and its later amendments.

A 55-year-old woman was admitted to our department with a 6-month history of abdominal distension and tea-colored urine, symptoms that had progressed during the week preceding admission and were accompanied by scleral icterus. She had been infected with HBV for over 30 years, though details regarding the mode of transmission, initial presentation, historical viral load, and prior liver function were unavailable. The patient had not received regular antiviral treatment (e.g., nucleos(t)ide analogues or interferon) and had not undergone guideline-recommended surveillance, including periodic assessment of liver function, HBV-deoxyribonucleic acid levels, alpha-fetoprotein (AFP), or hepatic imaging. She occasionally self-administered unspecified hepatoprotective agents during symptomatic episodes without medical supervision.

**Figure 1.**
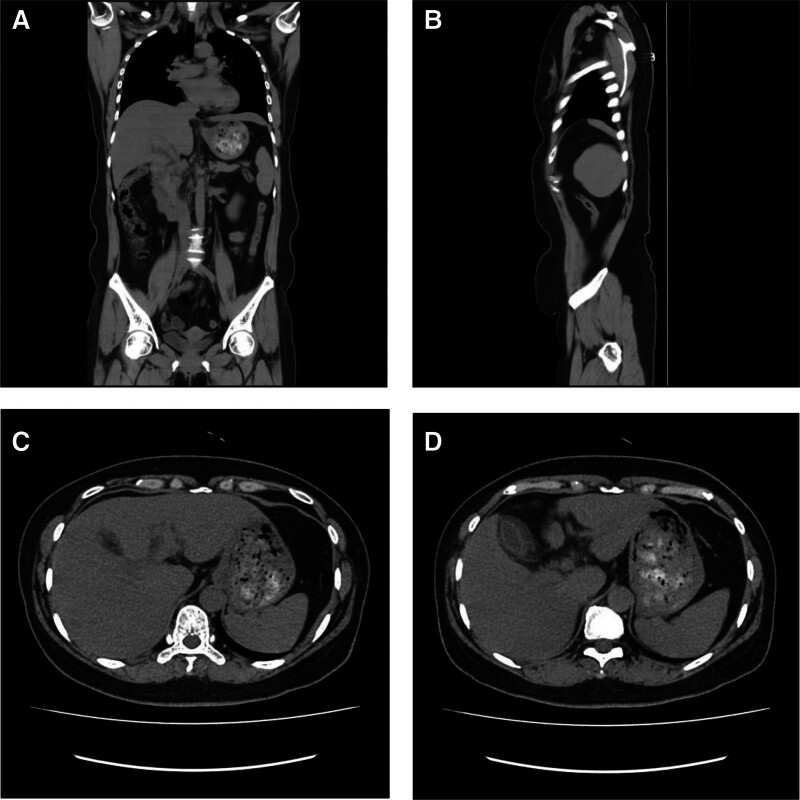
Abdominal plain CT. (a) Coronal. (b) Sagittal. (c, d) Axial. CT = computed tomography.

Her medical history was negative for chronic diseases, including hypertension, diabetes, and coronary artery disease, as well as for infectious diseases such as typhoid or tuberculosis. She reported no previous surgeries, trauma, blood transfusions, or allergies to medications or food. Vaccination status was unknown. The patient denied tobacco use, alcohol consumption (both chronic and intermittent), and high-risk sexual behavior. She described a routine lifestyle without significant exposure to toxins or chemicals, although potential occupational exposure could not be fully assessed due to lack of occupational details. There was no family history of inherited disorders. At admission, vital signs were stable: body temperature 35.6 °C, blood pressure 116/76 mm Hg, and heart rate 79 bpm. Physical examination revealed mild jaundice but no hepatomegaly or tenderness over the right upper quadrant. Contrast-enhanced abdominal CT demonstrated diffuse thickening of the gallbladder wall, consistent with adenomyomatosis (Fig. 1). Key laboratory results obtained during hospitalization are summarized below:

Time-stamped laboratory evolution:

Day 0 (May 05, 2024)-admission:

- Alanine aminotransferase (ALT): 391.9 U/L (ref: 7–40 U/L)
- Aspartate aminotransferase (AST): 258.3 U/L (ref: 13–35 U/L)
- Total bilirubin: 79.6 μmol/L (ref: 0–21 μmol/L)
- Direct bilirubin: 50.3 μmol/L (ref: 0–4 μmol/L)
- Total bile acids: 208.3 μmol/L (ref: 0.1–10 μmol/L)
- AFP: 505.73 nanograms per milliliter (ng/mL) (ref: 0–9 ng/mL)
- CA19-9: 284.8 U/mL (ref: 0–37 U/mL)
- HBV-deoxyribonucleic acid: 6.96 × 10⁴ IU/mL (ref: <10 IU/mL)
- Urinalysis: Urobilinogen 1+, Bilirubin 1+

Day 4 of treatment (May 14, 2024):

- ALT: 143.1 U/L (64% reduction from admission)
- AST: 106.3 U/L (59% reduction from admission)
- Total bilirubin: 48.9 μmol/L (39% reduction from admission)
- Direct bilirubin: 29.6 μmol/L (41% reduction from admission)
- Total bile acids: 98.6 μmol/L (53% reduction from admission)
- AFP: 724.95 ng/mL (43% increase from admission)
- CA19-9: 99.0 U/mL (65% reduction from admission)
- Urinalysis: Urobilinogen 1+, Bilirubin 1+

Day 8 of treatment (May 18, 2024):

- ALT: 50.1 U/L (87% reduction from admission)
- AST: 58.1 U/L (78% reduction from admission)
- Total bilirubin: 29.9 μmol/L (62% reduction from admission)
- Direct bilirubin: 19.4 μmol/L (61% reduction from admission)
- Total bile acids: 61.5 μmol/L (70% reduction from admission)
- AFP: 778.22 ng/mL (54% increase from admission)
- CA19-9: 51.6 U/mL (82% reduction from admission)

Additional parameters (single measurement at admission):

- Cyfra 21-1: 6.31 ng/mL (ref: 0.00–3.30 ng/mL)
- Serum amylase: 85 U/L (ref: 35–135 U/L)
- Urine amylase: 616.5 U/L (ref: 0–1200 U/L)
- Lipase: 77.9 U/L (ref: 23–300 U/L)
- Coagulation profile: Within normal limits
- Glucose: Not determined

**Figure 2.**
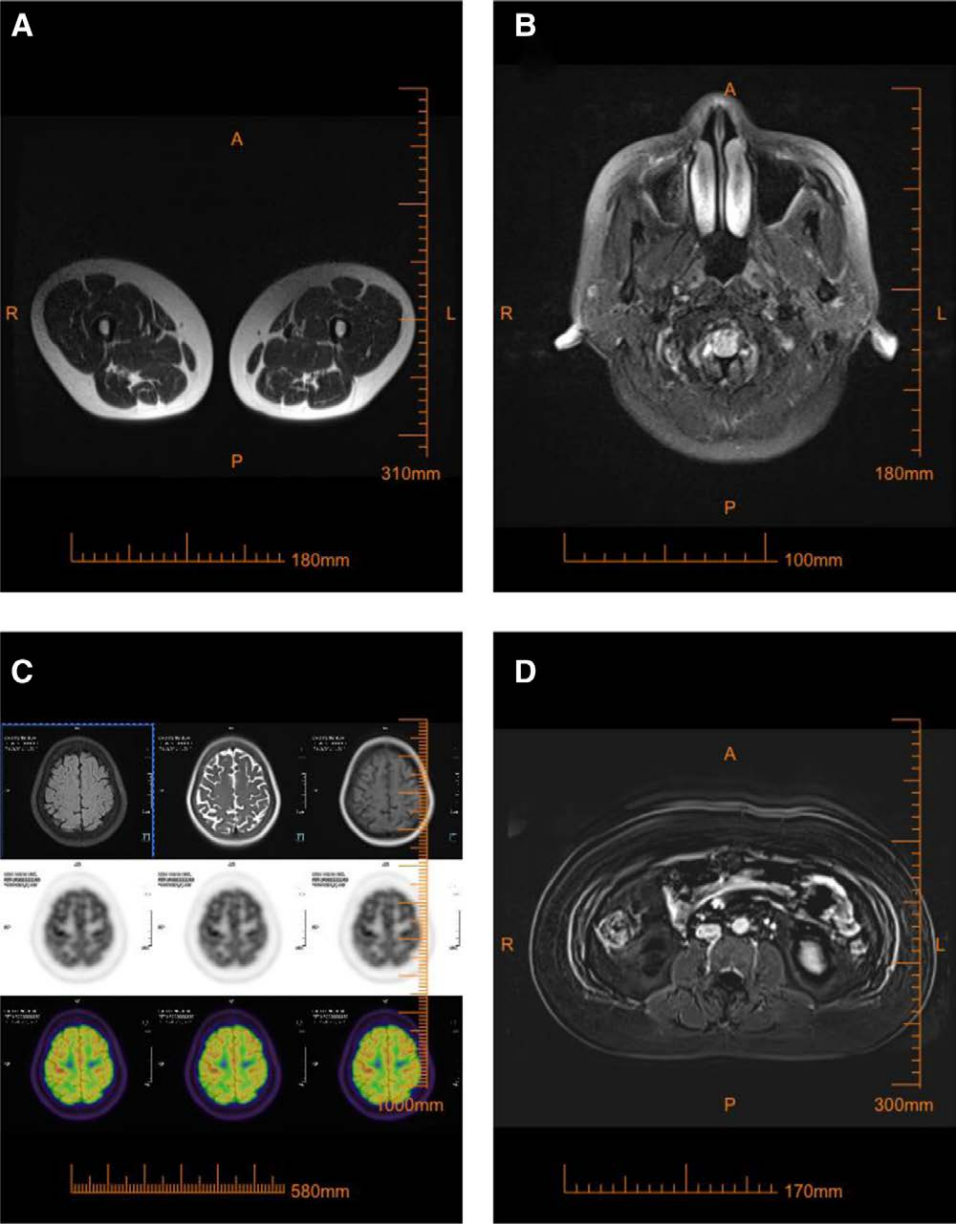
Whole-body PET-MRI. (a) Sagittal. (b) Axial. (c, d) Coronal. PET-MRI = Positron emission tomography–magnetic resonance imaging.

**Figure 3.**
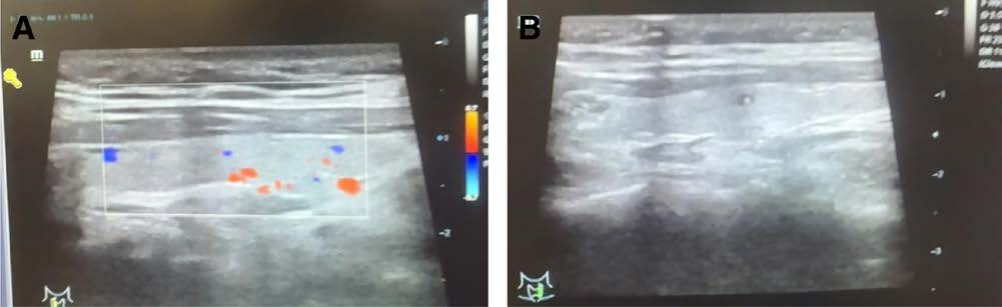
Thyroid color Doppler ultrasound. (a) Right lobe. (b) Left lobe.

**Figure 4.**
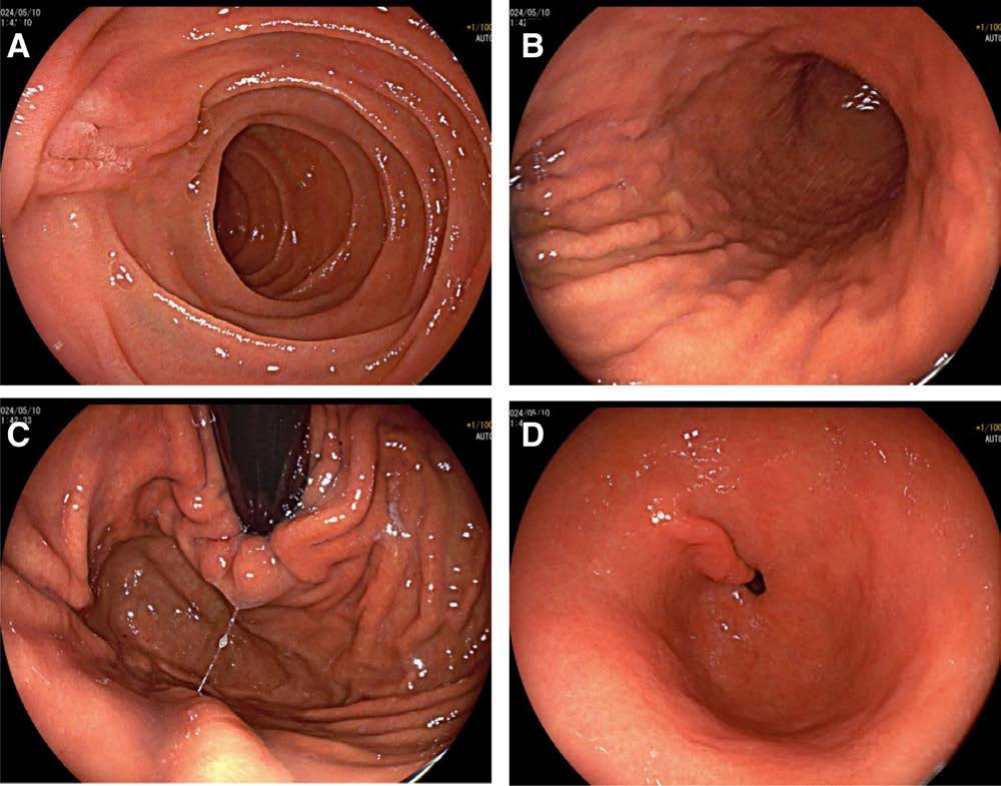
Upper gastrointestinal endoscopy. (a) Descending duodenum. (b) Gastric body. (c) Gastric fundus. (d) Gastric antrum.

**Figure 5.**
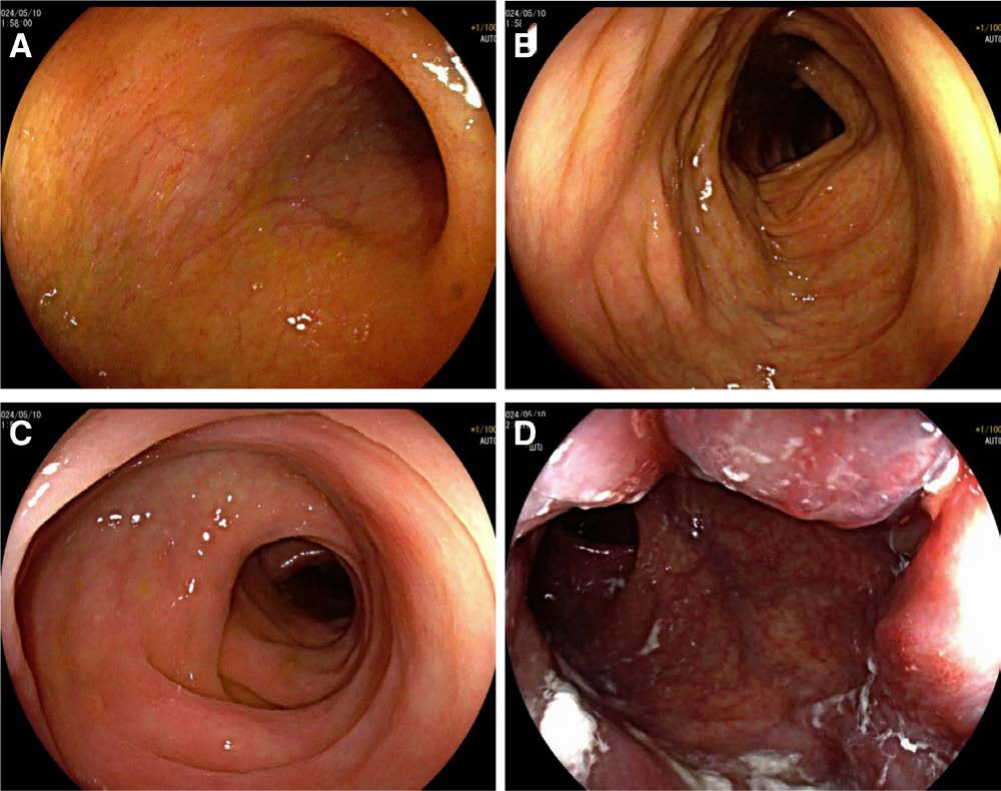
Colonoscopy. (a) Terminal ileum. (b) Transverse colon. (c) Descending colon. (d) Anal orifice.

The patient demonstrated rapid improvement in hepatic injury and cholestasis parameters within the first week of treatment, with aminotransferases and bilirubin showing progressive decline. However, the tumor markers exhibited divergent dynamics during clinical recovery: AFP levels rose by ~54%, whereas CA19-9 kinetics showed a striking decline of over 80% that correlated with the resolution of cholestasis. This discordant tumor marker profile warrants further investigation for potential hepatocellular carcinoma. Notably, the patient’s serum CA19-9 level was significantly elevated at 284.8 U/mL (reference range: 0–37 U/mL). Given the clinical necessity to exclude malignancy and following thorough informed consent obtained from the patient, a whole-body Positron emission tomography-MRI examination was promptly arranged. The Positron emission tomography-MRI findings (Fig. 2) revealed multiple abnormalities, including a suspected pituitary lesion, gallbladder polyps, chronic cholecystitis, a right breast lower quadrant cyst, hepatic steatosis, inflammatory lymph node hyperplasia in the portocaval and retroperitoneal regions, Nabothian cysts, a sacral canal cyst, and slightly reduced thyroid lobe size with heterogeneous signal intensity without hypermetabolism. Subsequent thyroid ultrasound (Fig. 3) demonstrated multiple colloid cysts (Chinese Thyroid Imaging Reporting and Data System 2) in the left lobe and an unremarkable right lobe and isthmus (Chinese Thyroid Imaging Reporting and Data System 2). Upper and lower gastrointestinal endoscopies showed no malignant features; gastroscopy (Fig. 4) revealed chronic superficial gastritis, with histopathology confirming mild chronic inflammation with intestinal metaplasia and polypoid hyperplasia. Colonoscopy (Fig. 5) was normal except for internal hemorrhoids. The patient was diagnosed with hepatocellular jaundice based on comprehensive laboratory and multimodal imaging findings, with no radiological evidence of pancreatic or other malignancies. Management followed established clinical guidelines and incorporated etiology-specific and supportive measures, including: hepatoprotection with intravenous glutathione and magnesium isoglycyrrhizinate, combined with oral Wuzhi Soft Capsules and Liver Protection Tablets; jaundice alleviation using Babao Dan Capsules (clearing dampness–heat and detoxifying) and Yinzhihuang Granules (promoting gallbladder function and reducing jaundice); supportive management with sucralfate suspension, Bacillus licheniformis capsules, trimebutine maleate, and esomeprazole; and antiviral therapy with entecavir. Following treatment, the patient’s CA19-9 levels normalized and gastric histopathology revealed no malignancy. Throughout the course of treatment, the patient’s jaundice symptoms improved progressively, accompanied by a marked decline in serum CA19-9 levels. During hospitalization, the CA19-9 level decreased to 99 U/mL. By the time of discharge, skin and scleral jaundice had significantly resolved, and jaundice-associated symptoms, including pruritus, had completely subsided. Repeat measurement of CA19-9 at discharge showed a further reduction to 51.6 U/mL. At the 6-month outpatient follow-up, the CA19-9 level had returned to within the normal range, measuring 35.9 U/mL.

## 3. Discussion

This report describes an uncommon clinical presentation of markedly elevated CA19-9 levels in the context of HBV-related hepatocellular jaundice and explores its potential mechanisms and clinical significance. Our investigation elucidates the pathophysiological relationship between HBV infection and increased CA19-9 levels, challenging the conventional paradigm that restricts CA19-9 application to malignancy detection. The results indicate that CA19-9 may serve as a valuable inflammatory biomarker for monitoring HBV-related hepatic injury, thereby establishing a new clinical rationale for its application in the assessment of benign liver diseases.

The pathophysiology of hepatocellular jaundice is centered on disrupted bilirubin metabolism due to hepatocyte damage, which subsequently leads to abnormal accumulation of bilirubin in the bloodstream.^[16]^ Physiologically, bilirubin metabolism relies on a coordinated hepatic process involving uptake, conjugation, and excretion. Impairment of hepatocyte function disrupts each of these steps, leading to diminished bilirubin clearance. Consequently, unconjugated bilirubin accumulates systemically, while cellular swelling and necrosis permit conjugated bilirubin to reflux into the circulation. Altogether, these mechanisms underlie the hyperbilirubinemia characteristic of hepatocellular jaundice.^[17]^ HBV infection is a major etiological factor in hepatocellular jaundice, operating through 3 interconnected mechanisms: viral replication induces immune-mediated hepatocyte inflammation and necrosis; subsequent hepatocellular dysfunction impairs bilirubin-metabolizing enzymes and transport proteins, disrupting metabolic balance; and HBV-related inflammation damages intrahepatic bile ducts, promoting cholestasis and establishing a self-perpetuating cycle of worsening jaundice.^[18,19]^ Diagnosis was based on integrated clinical, laboratory, and imaging findings. The patient presented with classic jaundice symptoms, abdominal distension, tea-colored urine, and scleral icterus, against a background of chronic HBV infection with high viral replication, supporting HBV-associated hepatocellular injury. Laboratory results revealed conjugated and unconjugated hyperbilirubinemia, markedly elevated aminotransferases, and urinary bilirubin, consistent with hepatocellular jaundice.^[20]^ Abdominal CT showed no biliary dilation, excluding obstructive jaundice; normal hematologic and hemolytic parameters ruled out hemolytic jaundice. Although tumor markers (CA19-9 and AFP) were elevated, the absence of malignant lesions on imaging and subsequent normalization of CA19-9 posttreatment argued against malignancy. The diagnosis was confirmed by significant clinical and biochemical improvement following antiviral and supportive therapy.

The elevated CA19-9 level in this patient was primarily driven by high-load HBV infection, with a potential contributory role from concomitant *H pylori* infection. The underlying mechanisms involve inflammation-induced hypersecretion, wherein HBV-triggered immune activation and biliary inflammation stimulate CA19-9 release from ductal epithelial cells^[20]^; biliary excretion^[21]^ impairment due to cholestasis and epithelial pressure; disrupted hepatic metabolic clearance resulting from HBV-induced liver dysfunction; and immune-mediated dysregulation of synthesis via pro-inflammatory cytokines (e.g., tumor necrosis factor-alpha, interleukin-6). Additionally, *H pylori*-associated gastritis may promote aberrant CA19-9 secretion through inflammatory activation of the gastroduodenobiliary epithelium. The observed reduction in CA19-9 following antiviral therapy underscores the predominant role of HBV, while the potential contribution of *H pylori* warrants further evaluation. Furthermore, the aberrant tumor markers in this case require careful clinical interpretation. The elevated AFP level can be attributed to hepatocellular regeneration rather than malignancy. Patients with chronic hepatitis B or cirrhosis often exhibit increased AFP levels during active hepatic repair, with concentrations correlating positively with the intensity of regenerative activity.^[22]^ In this case, the long-term HBV infection and high viral load indicate ongoing inflammatory injury and compensatory hepatocyte proliferation, supporting a benign etiology for the AFP elevation. Regarding the elevated cytokeratin 19 fragment, this marker lacks specificity and may be mildly elevated in various nonmalignant conditions, including hepatobiliary inflammation and gastrointestinal diseases. The absence of respiratory symptoms and normal chest imaging ruled out pulmonary malignancy, suggesting that the cytokeratin 19 fragment elevation likely resulted from systemic inflammation associated with HBV infection.

Furthermore, the observed elevation of CA19-9 in this case is more consistent with a paraneoplastic phenomenon driven by robust hepatic inflammation rather than an underlying malignancy. It is well-established that chronic inflammatory states, particularly in the liver, can lead to the aberrant production and release of various glycoproteins, including biomarkers typically associated with cancer. This process is often mediated by a complex network of pro-inflammatory cytokines. In the context of HBV-related liver injury, the persistent immune activation creates a microenvironment conducive to the upregulation of such cytokines, which can, in turn, stimulate the expression of glycoproteins such as CA19-9 in the biliary and hepatic epithelium. Recent research^[23]^ provides a compelling parallel: Chen et al (2025) demonstrated that a botanical extract could ameliorate metabolic disorder by specifically targeting and suppressing the NLRP3 inflammasome, a key driver of inflammation that leads to the maturation and secretion of potent cytokines such as IL-1β and IL-18. The fact that a purely inflammatory intervention can systemically alter metabolic and biomarker profiles underscores a fundamental principle: inflammation alone is a sufficient trigger for biomarker dysregulation. Therefore, by analogy, the severe inflammatory milieu in this patient’s liver, akin to the model described by Chen et al, provides a plausible and self-consistent explanation for the CA19-9 elevation, without necessitating the presence of an occult tumor.

This case provides 3 essential insights for clinical practice: a cognitive shift is needed to move beyond the misconception that CA19-9 elevation specifically indicates malignancy, as it can also markedly increase in benign conditions such as HBV-related hepatocellular jaundice, often correlating with inflammatory activity, as demonstrated in this case where high-load HBV infection led to a significant rise in CA19-9 through multiple mechanisms, underscoring the importance of recognizing even substantial elevations in the absence of cancer; in terms of patient management, a structured approach encompassing etiological investigation – including detailed medical and family history, viral and tumor marker profiling, and abdominal/chest imaging – followed by treatment monitoring with weekly laboratory assessments for 2 to 4 weeks to track biomarker trends, and long-term follow-up with quarterly to semiannual evaluations of viral load, liver function, and imaging to detect potential malignant transformation or disease progression; and regarding risk mitigation, clinicians should be aware of the dangers of overreliance on CA19-9 leading to overtreatment, the propensity of high-load HBV to advance to cirrhosis or hepatocellular carcinoma requiring continuous antiviral therapy and monitoring, and the risk of chronic cholestasis and biliary complications necessitating regular biliary imaging.

Future research should focus on 3 key directions to further elucidate the clinical relevance of CA19-9 in benign liver diseases: first, to validate the correlation between HBV viral load and CA19-9 levels through larger cohort studies and evaluate its potential as a quantitative biomarker for assessing the severity of HBV-related liver injury; second, to characterize CA19-9 expression patterns in other viral hepatitis-related jaundice, such as hepatitis C virus infection, in order to establish a more comprehensive framework for its application in benign hepatobiliary conditions; third, to investigate the utility of CA19-9 in evaluating anti-inflammatory treatment efficacy based on its association with inflammatory cytokines, thereby expanding its dual value in both clinical practice and scientific research.

## 4. Conclusions

In typical presentations of hepatocellular jaundice, CA19-9 levels generally remain within normal limits and do not exhibit dynamic fluctuations correlating with disease activity. We describe a case of hepatocellular jaundice in which a significantly elevated CA19-9 level was observed in the absence of malignant disease. Notably, the CA19-9 level decreased concurrently with the resolution of jaundice following treatment, eventually normalizing during recovery. This temporal association suggests that CA19-9 may serve as an ancillary biomarker reflecting disease severity and treatment response in certain cases of hepatocellular jaundice. Although further case accumulation is necessary to establish its clinical utility, this phenomenon highlights that even markedly elevated CA19-9 levels may occur in benign conditions. A mild-to-moderate elevation in CA19-9 could thus provide additional diagnostic clues in the evaluation of hepatocellular jaundice.

## Author contributions

**Writing – original draft:** Yangqing Cui.

**Writing – review & editing:** Yangqing Cui, Yijing Zhang, Yuxi Huang, Rui Feng, Qunqun Chen.
